# Supplementary material for: Cognitive Impairment in Convalescent COVID-19 Patients Undergoing Multidisciplinary Rehabilitation: The Association with the Clinical and Functional Status
Source: Healthcare (Basel). 2022 Mar 4;10(3):480. doi: 10.3390/healthcare10030480 (PMC8950669; doi:10.3390/healthcare10030480)
Supplement: Supplementary file 1 [file healthcare-10-00480-s001.zip › healthcare-1593088-supplementary.pdf]

Supplementary online material

Cognitive Impairment in Convalescent COVID-19 Patients Undergoing Multidisciplinary Rehabilitation: The Association with the Clinical and Functional Status

Pasquale Moretta [1,*,†], Pasquale Ambrosino [2,*,†], Anna Lanzillo [1], Laura Marcuccio [1], Salvatore Fuschillo [3], Antimo Papa [2], Gabriella Santangelo [4], Luigi Trojano [4,‡] and Mauro Maniscalco [3,‡]

[1] Istituti Clinici Scientifici Maugeri IRCCS, Neurological Rehabilitation Unit of Telese Terme Institute,
82037 Telese Terme, Italy; anna.lanzillo@icsmaugeri.it (A.L.); laura.marcuccio@icsmaugeri.it (L.M.)

[2] Istituti Clinici Scientifici Maugeri IRCCS, Cardiac Rehabilitation Unit of Telese Terme Institute, 82037 Telese Terme, Italy; antimo.papa@icsmaugeri.it

[3] Istituti Clinici Scientifici Maugeri IRCCS, Pulmonary Rehabilitation Unit of Telese Terme Institute,
82037 Telese Terme, Italy; salvatore.fuschillo@icsmaugeri.it (S.F.); mauro.maniscalco@icsmaugeri.it (M.M.)

[4] Department of Psychology, Università della Campania Luigi Vanvitelli, 81100 Caserta, Italy; gabriella.santangelo@unicampania.it (G.S.); luigi.trojano@unicampania.it (L.T.)

* Correspondence: pasquale.moretta@icsmaugeri.it (P.M.); pasquale.ambrosino@icsmaugeri.it (P.A.)

† The two Authors equally contributed to the manuscript.

‡ The two Authors share co-seniorship.

Table of contents

**Supplemental Table S3**	- **Clinical and demographical characteristics of the sample as a function of sex.**<br>- **Raw scores of neuropsychological tests.**<br>- **Correlations between measure of clinical, functional and cognitive status.**
**Supplemental Figure S1**	**Flow chart of study participants.**

Supplemental Figure S1. Flow chart of study participants.

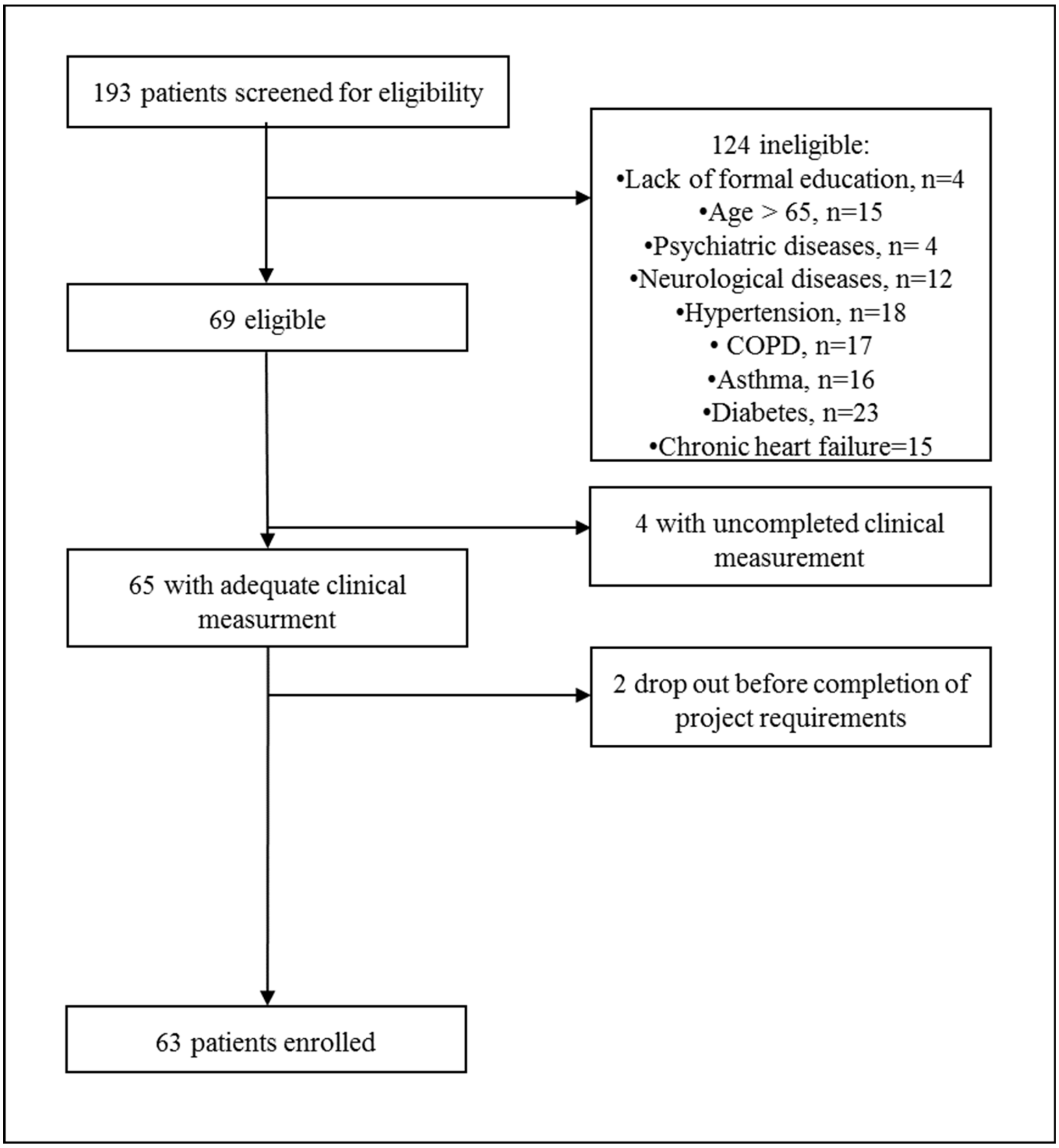

Supplemental Table S1. Clinical and demographical characteristics of the sample as a function of sex.

Demographical and Clinical measures/groups	Females (n= 16) mean (S.D.)	Males (n= 47) mean (S.D.)	z	p
**Age**	61.56 (10.191)	59.32 (11.311)	.787	.431
**Education (years)**	10.19 (3.710)	12.48 (3.427)	2.188	.029
**Disease duration (days)**	54.20 (35.86)	48.24 (25.86)	.642	.521
**Duration of hospital stay (days)**	30.88 (19.565)	23.68 (15.340)	1.225	.220
**Duration of bed stay (days)**	24.56 (25.076)	22.11 (15.731)	.247	.805
**D-Dimer (ng/ml)**	626.00 (391.368)	597.77 (587.138)	1.154	.248
**CRP (mg/l)**	6.586 (8.999)	10.161 (19.275)	.361	-.718
**PaO2 (mmHg)**	81.785 (14.515)	73.625 (14.822)	1.798	.072
**PaCO2 (mmHg)**	40.900 (13.893)	36.359 (6.537)	1.656	.098
**FEV1 (% predicted)**	77.62 (22.747)	79.21 (20.611)	.305	.760
**FVC (% predicted)**	76.46 (22.393)	76.09 (20.224)	.153	.879
**FEV1/FVC**	80.80 (7.59)	82.55 (7.65)	.617	.537
**DLCO (% predicted)**	44.88 (12.04)	60.74 (21.48)	2.108	.035
**DLCO/VA (% predicted)**	73.71 (19.88)	80.29 (22.07)	.858	.391
**Mean daytime SBP (mmHg)**	120.43 (23.43)	121.28 (12.86)	.604	.546
**Mean daytime DBP (mmHg)**	77.24 (12.15)	79.64 (8.77)	.899	.369
**Mean nighttime SBP (mmHg)**	117.65 (20.86)	116.07 (15.17)	.155	.877
**Mean nighttime DBP (mmHg)**	72.12 (11.02)	73.52 (9.68)	.449	.653
**HADS_Anxiety score**	10.64 (3.529)	8.96 (3.261)	1.468	.142
**HADS_Depression score**	11.45 (3.778)	11.90 (4.304)	.215	.830
**STAI - Trait score**	41.64 (11.893)	39.55 (8.129)	.708	.479
**BARTHEL score**	79.00 (23.664)	73.41 (25.942)	.817	.414
**EQ-5D Visual Analog Scale**	3.172 (10.258)	18.754 (32.287)	.585	.559

Abbreviations: CRP: C-reactive protein; PaO2: partial pressure of oxygen in arterial blood; PaCO2: partial pressure of carbon dioxide; FEV1: forced expiratory volume in 1 second; FVC: forced vital capacity; DLCO: diffusion lung capacity for carbon monoxide; VA: alveolar volume; SBP: systolic blood pressure; DBP: diastolic blood pressure; HADS: Hospital Anxiety and Depression Scale; STAI: State trait Anxiety Questionnaire (trait anxiety); EQ-5D: Euro QoL.

Supplemental Table S2. Raw scores of neuropsychological tests.

Neuropsychological tests	Total Sample (N= 63) Mean (S.D.)	RCE (n= 28) Mean (S.D.)	NCE (n= 35) Mean (S.D.)	p
**Montreal Cognitive Assessment**	25.24 (2.55)	23.43 (3.51)	26.78 (2.16)	.016
**Frontal Assessment Battery**	16.28 (1.09)	15.93 (.979)	16.56 (1.10)	.029
**Coloured Proggressive Matrix**	28.41 (4.50)	27.11 (3.76)	29.42 (4.81)	.046
**Verbal Fluency (FAS)**	29.50 (10.24)	25.57 (9.90)	32.56 (9.56)	**.004**
**Trail Making Test (B-A)**	79.79 (50.56)	109.71 (48.71)	58.68 (40.65)	**.000**
**RAVLT – immediate recall**	42.17 (9.31)	34.00 (8.87)	51.97 (19.32)	**.001**
**RAVLT – delayed recall**	7.67 (3.02)	6.33 (3.23)	8.67 (2.44)	**.002**
**Corsi's Test**	4.88 (.92)	4.32 (.72)	5.30 (.84)	**.000**
**Corsi's supra-span Corsi's Test**	10.09 (1.23)	7.41 (.98)	12.78 (1.11)	**.000**

Abbreviations: Corsi's supra-span: Supra-span learning on Corsi's Test; RAVLT: Rey's Auditory Verbal Learning Test. In bold are reported the significant differences according to Bonferroni's correction (p value= .005; for 9 comparisons).

Supplemental Table S3. Correlations between measure of clinical, functional and cognitive status.

	1	2	3	4	5	6	7	8	9	10	11	12	13	14
1. Hospital Stay duration	1													
2. Bed rest duration	.712**	1												
3. HADS-A	-.106	-.084	1											
4. HADS-D	-.039	-.031	.204	1										
5. NPSCT	.047	.112	.193	.252	1									
6. EQ-5D	-.132	-.149	-.130	.126	-.263	1								
7. PaO2	-.342**	-.457**	.023	-.166	-.264*	.223	1							
8. PaCO2	.149	-.077	.017	-.203	-.048	.053	-.583**	1						
9. FEV1%	-.224	-.312*	-.086	-.267	-.156	.223	.578**	.069	1					
10. FVC%	-.337**	-.335**	-.068	-.287*	-.173	.193	.579**	-.114	.945**	1				
11. FEV1/FVC%	-.072	-.035	.122	.078	.069	.257	-.069	.174	.074	-.241	1			
12. DLCO%	-.161	-.336*	.078	-.050	-.351*	-.062	.501**	.139	.657**	.661**	-.030	1		
13. DLCO/VA%	-.199	-.145	.258	.228	-.188	.134	.106	.126	.147	.062	.380	.558**	1	
14. ACRBP	.062	.205	-.039	.066	.410**	-.186	-.180	-.096	-.144	-.069	-.161	-.132	-.069	1

HADS: Hospital Anxiety (A) and Depression (D) Scale; NPSCT: number of pathological scores on cognitive test; EQ-5D: Euro QoL; Pao2: partial pressure of oxygen in arterial blood; PaCO2: arterial partial pressure of carbon dioxide; FEV1%: forced expiratory volume in 1 second; FVC: forced vital capacity; DLCO: diffusion lung capacity for carbon monoxide; VA: alveolar volume; ACRBP: altered circadian rhythm of blood pressure. Spearman's coefficients: *asterisk indicates p value <0.05; **indicates p value <0.01.
